# Dynamic hip kinematics during squatting before and after total hip arthroplasty

**DOI:** 10.1186/s13018-018-0873-3

**Published:** 2018-07-03

**Authors:** Keisuke Komiyama, Satoshi Hamai, Daisuke Hara, Satoru Ikebe, Hidehiko Higaki, Kensei Yoshimoto, Kyohei Shiomoto, Hirotaka Gondo, Yifeng Wang, Yasuharu Nakashima

**Affiliations:** 1 Department of Orthopedic Surgery, Graduate School of Medical Sciences, Kyushu University, 3-1-1 Maidashi, Higashi-ku, Fukuoka, 812-8582 Japan; 2 Department of Life Science, Faculty of Life Science, Kyushu Sangyo University, 2-3-1 Matsugadai, Higashi-ku, Fukuoka, 813-0004 Japan; 3 Department of Creative Engineering, National Institute of Technology, Kitakyushu College, 5-20-1 Shii, Kokuraminami-ku, Kitakyushu, Fukuoka, 802-0985 Japan

**Keywords:** Kinematics, Range of motion, Squatting, Total hip arthroplasty, 3D-to-2D model-to-image registration techniques

## Abstract

**Background:**

The difference in in vivo kinematics before and after total hip arthroplasty (THA) for the same subjects and the clearance between the liner and neck during squatting have been unclear. The purpose of the present study was to clarify (1) the changes in the in vivo kinematics between prosthetic hips and osteoarthritis hips of the same subjects and (2) the extent of the liner-to-neck clearance during squatting under weight-bearing conditions.

**Methods:**

This study consisted of 10 patients who underwent unilateral THA for symptomatic osteoarthritis. Using a flat-panel X-ray detector, we obtained continuous radiographs during squatting. We analyzed the hip joint’s movements using three-dimensional-to-two-dimensional model-to-image registration techniques. We also quantified the minimum distance at maximum flexion and extension, and the minimum angle at maximum flexion between the liner and stem neck.

**Results:**

The maximum hip flexion angles post-THA (80.7° [range, 69.4–98.6°]) changed significantly compared with the pre-THA values (71.7° [range, 55.2°–91.2°]). The pelvic tilt angle (posterior +, anterior−) at the maximum hip flexion post-THA (10.4° [range, − 6.7° to 26.9°]) was significantly smaller than that at pre-THA (16.6° [range, − 3° to 40.3°]). The minimum anterior and posterior liner-to-neck distances averaged 10.9 and 8.0 mm, respectively, which was a significant difference. The minimum liner-to-neck angle at maximum flexion averaged 34.7° (range, 20.7°–46.3°). No liner-to-neck contact occurred in any of the hips.

**Conclusion:**

THA increased the range of hip joint motion and the pelvis tilted anteriorly more after than before THA, with sufficient liner-to-neck clearance during squatting. These data may be beneficial for advising patients after THA regarding postoperative activity restrictions in daily life.

## Background

Total hip arthroplasty (THA) is the best surgical procedure for patients with end-stage osteoarthritis (OA) of the hip joint and is highly effective in relieving pain and improving function [1–3]. The clinical success of THA allows patients to resume their activities of daily living easily, and this includes squatting motions [4]. In Non-Western cultures, squatting is one of the fundamental activities of daily living and requires deep ranges of motion in flexion [5, 6]. Due to such requirements for deep flexibility of the prosthetic hip, there are concerns for impingement and dislocation after THA. Therefore, the knowledge of the in vivo kinematics of the prosthetic hip associated with squatting and the clearance between the liner and neck (liner-to-neck clearance) could be beneficial for advising for patients after THA regarding postoperative activity restrictions in daily life.

Accurate evaluations of kinematics under weight-bearing conditions have been achieved using three-dimensional (3D)-to-two-dimensional (2D) model-to-image registration techniques [7–9]. Recently, these techniques have been applied to the kinematic analyses of hips affected by OA and subsequent THA procedures [10, 11]. We previously reported that, with respect to squatting, patients with OA were unable to deeply flex their femurs due to limited range of motion (ROM) of the hip joints. Additionally, patients with OA tilted their pelvis more posteriorly to maintain a deeply flexed posture than did healthy subjects [10]. In prosthetic hips, Koyanagi et al. revealed that the mean maximum hip flexion ROM was 86.2 °, which is smaller than the mean maximum hip flexion angle of 95.4 ° in normal native hips [5, 12]. However, to the best of our knowledge, no previous report has demonstrated the changes in in vivo 3D kinematics during squatting pre- and post-THA for the same subjects using the accurate 3D-to-2D model-to-image registration techniques.

The purpose of the present study was to clarify (1) the changes in the in vivo kinematics between prosthetic hips and OA hips of the same subjects and (2) the extent of the liner-to-neck clearance during squatting under weight-bearing conditions.

## Methods

### Patients

The protocol in the current study was approved by our institutional review board. All patients provided informed consent to participate in this study. Between August 2012 and October 2015, 177 patients with 200 hips underwent primary cementless THA by two senior surgeons (Y.N. and S.H.). Among the original 177 patients, 10 of them (10 hips) met all the inclusion criteria, which were (1) unilateral THA for symptomatic OA (2) no previous surgery of the ipsilateral hip, (3) no previous surgery of the spine or other joints, (4) obtaining the informed consent prior to THA, and (5) ability to squat safely without assistance before and after THA. Demographic data of the patients are shown in Table 1. There were 6 women and 4 men with a mean age at the time of THA of 65 ± 8 years (range, 55–84 years). The mean height was 156 ± 7 cm (range, 148–168 cm), and the mean body mass index (BMI) was 23 ± 4 kg/m^2^ (range, 18–31 kg/m^2^). According to the Kellgren-Lawrence scale, 2 hips were classified as grade III and 8 hips were classified as grade IV. [13] The pre- and post-THA mean Harris hip scores were 48 ± 7 (range, 40–57) and 95 ± 3 (range, 91–99), respectively. The mean post-THA follow-up was 43 ± 11 months (range, 23–59 months). No patient had a history of any complication after THA.

**Table 1:** Demographic data

Patient no.	1	2	3	4	5	6	7	8	9	10
Sex	Male	Male	Female	Female	Male	Female	Female	Female	Female	Male
Diagnosis	OA	DDH	DDH	DDH	OA	DDH	DDH	DDH	DDH	OA
Age at THA (years old)	61	65	56	60	55	70	68	63	68	84
Affected side	Right	Left	Left	Left	Right	Right	Left	Right	Right	Left
Height (cm)	155	161	161	148	168	153	148	152	149	163
Body weight (kg)	73	63	46	46	70	44	40	55	64	65
BMI (kg/m^2^)	30.5	24.3	17.7	21	24.7	18.8	18.3	24	28.5	24.5
Preoperative HHS	51	53	44	51	40	57	45	41	40	56
Postoperative HHS	97	98	91	91	91	98	94	99	97	95
Follow-up period (months)	59	47	49	38	49	36	36	23	48	48

*OA* osteoarthritis, *DDH* developmental dysplasia of the hip, *THA* total hip arthroplasty, *BMI* body mass index, *HHS* Harris hip score

### Surgical procedures and implants

All operations were performed through a posterolateral approach with repair of the posterior soft tissue [14]. The combined anteversion technique was adopted to cope with the wide range of femoral anteversion of hip dysplasia at our institution. The cup was placed according to stem anteversion so that combined anteversion ranged from 40° to 60° [15]. A cementless hemispherical press-fit cup, straight metaphyseal fit stem, and high cross-linked ultra-high molecular weight polyethylene liner (AMS and PerFix HA; Aeonian; Kyocera, Kyoto, Japan) were used [16, 17]. All femoral heads were alumina ceramic, and the head sizes were 32 mm in 9 cases and 26 mm in 1 case. There are two types of rims in the Aeonian liner, one is a 15 ° elevated rim (elevated rim liner) to prevent dislocation of the femoral head, and the other is not elevated (flat liner). Four hips used the elevated rim liners, and 6 hips used the flat liner, respectively. When using the elevated rim liner, we recorded the location on the cup where the top of the rim was placed.

The orientations of the acetabular cup and stem were measured using the postoperative computed tomography (CT) data. The cup inclination was measured as the angle of abduction using the inter-tear-drop line as the baseline (radiographic inclination). The cup anteversion was measured as the angle of anteversion in the sagittal plane (operative anteversion). Femoral anteversion was measured as the angle of anteversion between the prosthetic femoral neck and transepicondylar axis [18].

### Kinematic analysis

**Fig. 1:**
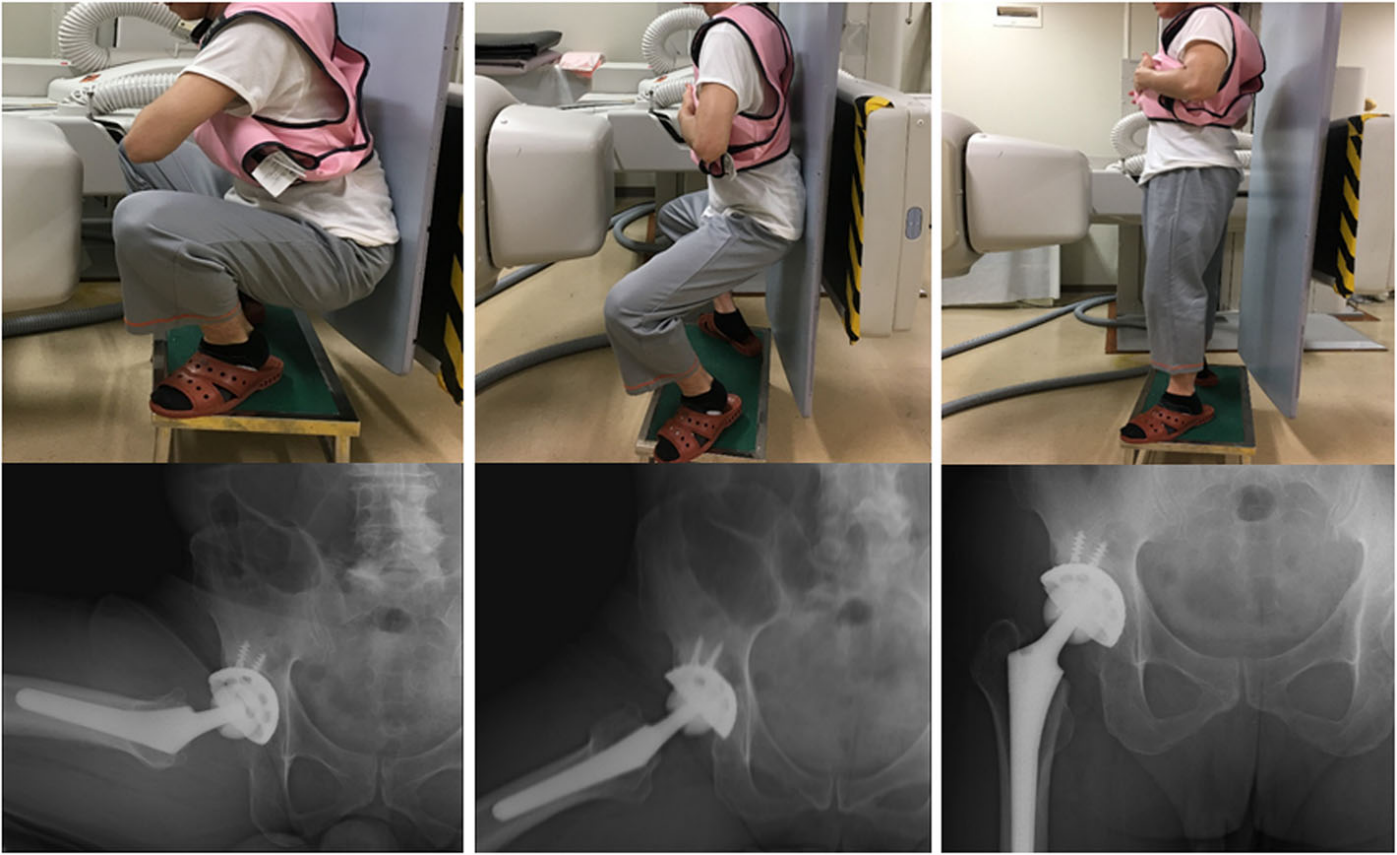
The hip motions during squatting were captured as continuous X-ray images using a flat panel X-ray detector. Patients before and after total hip arthroplasty stood from a squat position with their heel down

We essentially followed the method in accordance with previous reports, and we partially referenced the kinematics data regarding pre-THA [10, 19]. The 3D positions and orientations of the pelvis, acetabular cup, femur, and femoral stem during squatting were determined using 3D-to-2D model-to-image registration techniques. Continuous radiographic images were used to survey squatting movements using a flat-panel X-ray detector (Ultimax-I, Toshiba, Tochigi, Japan) with the following parameters: image area of 420 mm × 420 mm, resolution of 0.274 mm × 0.274 mm/pixel, and frame rate of 3.5 frames/s (Fig. 1). Each subject routinely underwent CT (Aquilion, Toshiba, Tochigi, Japan) with a 512 × 512 image matrix, a 0.35 × 0.35 pixel dim, and 1-mm-slice thicknesses from the superior edge of the pelvis to just below the knee joint line. Anatomical coordinate systems of the pelvis and femur were embedded in each bone model derived from CT data according to our previous study [10, 19]. Computer simulation was performed to generate virtual, digitally reconstructed radiographs in which the light source and the projected plane parameters were set to be identical to the actual radiographic imaging conditions. Each model silhouette was matched with the actual silhouette by translating and rotating the 3D model to minimize the number of unmatched pixels between the silhouettes. The orientation of the femur relative to the pelvis: hip movements, was determined using the Cardan/Euler angle system in *x*-*y*-*z* order (flexion/extension, adduction/abduction, internal/external rotation). The maximum errors associated with tracking the position of the femur/stem relative to the pelvis/acetabular cup were 0.36/0.43 mm, 0.37/0.48 mm, and 0.48 °/0.52 °, respectively, for in-plane translation, out-of-plane translation, and rotation, respectively [10, 19].

**Fig. 2:**
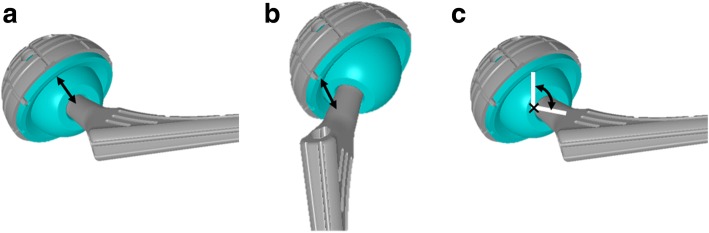
The minimum distance at maximum flexion (**a**) at maximum extension (**b**), and the angle at maximum flexion (**c**) between the liner and stem neck

Regarding the liner-to-neck clearance, we quantified the minimum distance at maximum flexion and extension and the angle at maximum flexion between the liner and stem neck using a computer-aided design software program ([CATIA V5]; Dassault Systemes, Vélizy-Villacoublay, France) (Fig. 2) [19, 20].

### Statistical analysis

All data are expressed as mean ± standard deviation (SD) and were tested for normality using the Shapiro-Wilk test. To compare hip kinematics before and after THA in the same patient, normally distributed variables were evaluated using the paired *t* test. Non-normally distributed variables were evaluated using the independent Wilcoxon signed-rank test. Statistical significance was defined as a *P* value < .05. The statistical analyses were performed using JMP Software (Version 11; SAS Institute, Cary, NC, USA).

## Results

### Orientation of the components

The mean cup inclination and mean cup anteversion were 39.1 ± 5.4° (range, 30.1°–48.0°) and 18.5 ± 12.0° (range, 4.3°–40.0°), respectively. The mean stem anteversion was 31.7 ± 6.8° (range, 22.0°–42.1°), and the mean combined anteversion was 50.3 ± 9.3° (range, 36.9°–67.5°) (Table 2).

**Table 2:** Component data

Patient no.	1	2	3	4	5	6	7	8	9	10
Cup size (mm)	52	50	48	46	52	48	48	48	50	54
Head size (mm)	32	32	32	26	32	32	32	32	32	32
Polyethylene liner	Flat	Flat	Flat	Flat	Elevated	Elevated	Flat	Flat	Elevated	Elevated
Cup inclination (°)	36.6	42	30.1	44	41.2	37.1	34.1	42.4	48	35.2
Cup anteversion (°)	4.3	16	11	10.1	26.8	5	24	35.3	38	14.9
Stem anteversion (°)	34.8	37	38.1	34	26.4	42.1	28.1	32.2	22.6	22
Combined anteversion (°)	39.1	53	49.1	44.1	53.2	47.1	52	67.5	60.6	36.9

### Kinematics of the hip joint

**Fig. 3:**
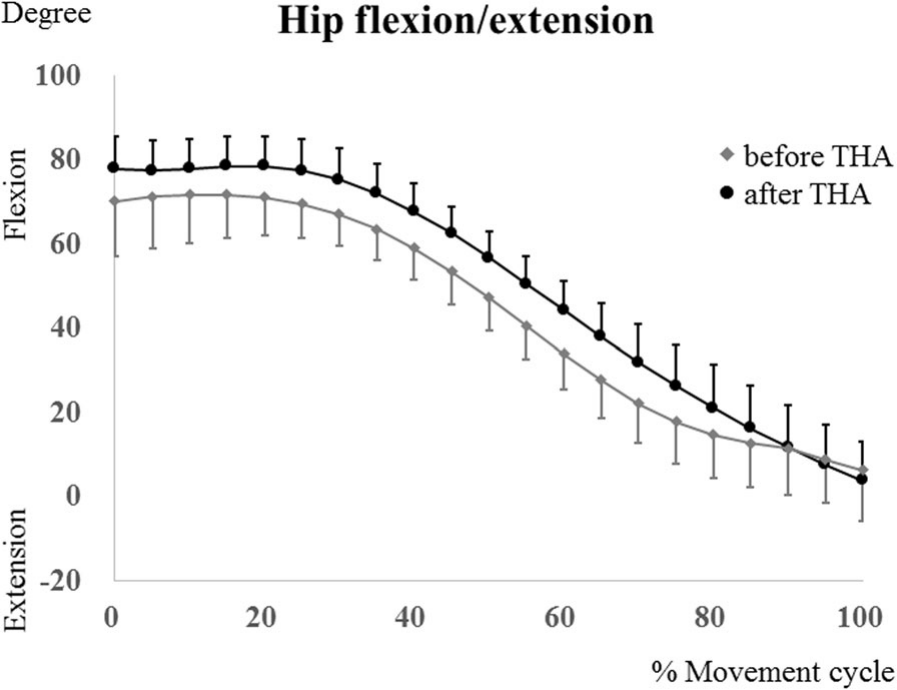
The hip flexion/extension angles during squatting in patients before total hip arthroplasty (THA) (gray lines) and patients after THA (black lines). Error bars show standard deviation

**Fig. 4:**
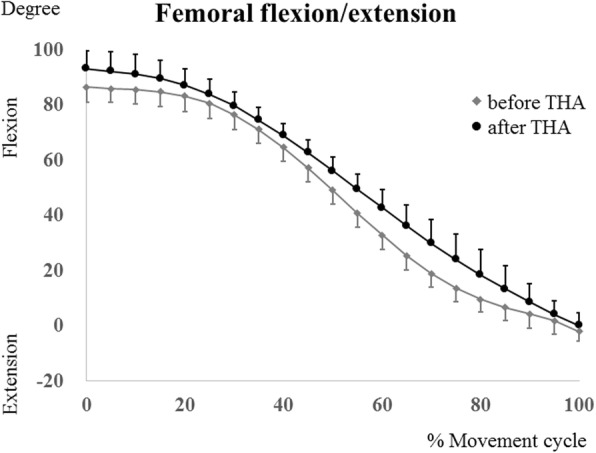
The femoral flexion/extension angles during squatting in patients before total hip arthroplasty (THA) (gray lines) and patients after THA (black lines). Error bars show standard deviation

**Fig. 5:**
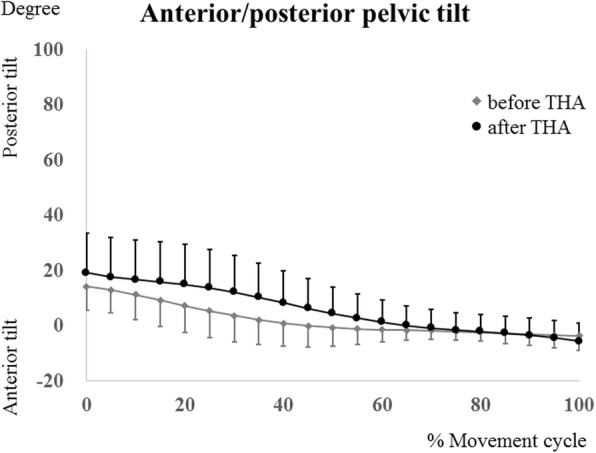
Posterior/anterior pelvic tilt angles [posterior +, anterior−] during squatting in patients before total hip arthroplasty (THA) (gray lines) and patients after THA (black lines). Error bars show standard deviation

The maximum hip flexion angles were determined during squatting at 10% of the squat ascent cycle pre-THA, and at 15% of the squat ascent cycle post-THA, respectively (Fig. 3). The kinematics data of each patient are shown in Table 3. The maximum femoral and hip flexion angles post-THA (94.9 ± 6.8° [range, 85.4°–104.6°] and 80.7 ± 10.0° [range, 69.4°–98.6°], respectively) changed significantly (*P* = .013 and *P* = .005, respectively) compared with the pre-THA values (86.2 ± 7.3° [range, 70.9°–94.3°] and 71.7 ± 11.9° [range, 55.2°–91.2°], respectively) (Figs. 3 and 4). In the squatting position, the pelvis tilted backward and gradually tilted forward with standing. The pelvic tilt angle (posterior +, anterior−) at the maximum hip flexion post-THA (10.4 ± 10.4° [range, − 6.7° to 26.9°] was significantly (*P* = .0463) smaller than that at pre-THA (16.6 ± 13.3° [range, − 3° to 40.3°]) (Fig. 5).

**Table 3:** Patient kinematic data

Patient No.	1	2	3	4	5	6	7	8	9	10
Maximum hip flexion (°)										
Pre-THA	59.5	73.1	67.2	83.5	73.3	70.7	59.4	84.3	91.2	55.2
Post-THA	75.6	73.3	71.6	90.5	81.3	87.5	71.2	87.6	98.6	69.4
Maximum femoral flexion (°)										
Pre-THA	77.2	92.9	85.8	93.8	87	86.9	87.2	85.5	94.3	70.9
Post-THA	96.5	92.1	91.7	102.1	86	92.1	104.6	85.4	103.1	95.1
Pelvic tilt at maximum hip flexion (°)										
Pre-THA	40.3	20.6	15.5	8.1	− 3	18.9	31.2	2.5	7.6	23.9
Post-THA	20.3	15.4	16.7	4.2	3.5	3.8	17.6	− 6.7	2.3	26.9
Liner-to-neck clearance										
the minimum anterior distance (mm)	10	9.5	11.4	8.2	11.2	12.4	12.4	12.1	14.8	7.3
the minimum posterior distance (mm)	10.1	7	9.9	8.9	11.2	7.3	10.1	5.8	6	3.6
the minimum angle at maximum hip flexion (°)	30.6	32.6	35.2	31.8	31	34.9	44	40.1	46.3	20.7

*THA* total hip arthroplasty

The pelvic tilt (posterior +, anterior−) angles

### Liner-to-neck clearance

The liner-to-neck clearance data for the each patient are shown in Table 3. The minimum anterior and posterior liner-to-neck distances averaged 10.9 ± 2.2 mm (range, 7.3–14.8 mm) and 8.0 ± 2.4 mm (range, 3.6–11.2 mm), respectively, and the difference was significant (*P* = .0143). The minimum liner-to-neck angle at maximum flexion averaged 34.7 ± 7.3° (range, 20.7–46.3°). No liner-to-neck contact occurred in any of the hips during squatting.

## Discussion

To our knowledge, no previous reports have demonstrated the changes in in vivo hip kinematics during squatting pre- and post-THA for the same subjects using accurate 3D-to-2D model-to-image registration techniques. The current study elucidated the dynamic hip kinematics during squatting before and after THA and quantified the liner-to-neck clearance. The maximum hip flexion significantly changed from 72° pre-THA to 81° post-THA, and the pelvic tilt angle at the maximum hip flexion post-THA was 10° , which was significantly smaller than the 17° measurement pre-THA. The anterior and posterior liner-to-neck distances averaged 11 and 8 mm, respectively, and the differences were significant. The mean minimum liner-to-neck angle was 35°, and there was no liner-to-neck contact during squatting in any of the hips.

A previous study found that patients with OA were not able to flex their femurs deeply due to limited ROM of the hip joints during squatting. They also tend to tilt their pelvises more posteriorly to maintain a deeply flexed posture than did healthy subjects [10]. The present study revealed that the maximum hip flexion was improved by approximately 9°. The pelvic tilt at the maximum hip flexion post-THA was significantly inclined and measured approximately 7° more anteriorly than pre-THA for the same subjects. We found that THA increased the range of the femoral and hip joint motions during squatting, and the compensation to maintain deeply flexed postures was reduced. Sagittal pelvic mobility is integral in flexing the torso to maintain balance and to allow the large hip flexion angles that are essential for deep squats [21].

It has been reported that approximately 95 to 102° of maximum hip flexion occurs during squatting in healthy subjects [5, 22]. Our study showed that the maximum hip flexion significantly changed from 72° pre-THA to 81° post-THA. Although THA provided increased ROM in patients with OA, prosthetic hips were not able to recover the kinematics to the level of healthy hips. Catelli et al. reported that joint kinematics of the pelvis and hip do not return to the level of healthy hips after THA during squatting and dual-mobility implant combined with the poorer functional scores [23]. A limited range of hip flexion persisted even after THA, which might have affected postoperative functional outcomes during activities of daily living, especially those requiring deeply flexed postures.

Specific postures might include potential risks of prosthetic impingement and could cause postoperative dislocation, and polyethylene wear after THA [24, 25]. However, we found only a few reports which quantified the liner-to-neck distances while squatting. In our study, the anterior and posterior liner-to-neck distances averaged 11 and 8 mm, respectively, and the differences were significant. Furthermore, the minimum liner-to-neck angle at maximum flexion was 21°. Koyanagi et al. reported that the minimum angle leading up to theoretical prosthetic impingement was more than 10° [12]. Based on the analysis, we concluded that the liner-to-neck clearance during squatting after THA was sufficient in these patients.

There are several limitations in the present study. First, the number of patients in this study was small. A larger sample size with a wider variation in component position might result in greater statistical reliability and reveal correlation between implant positioning and minimum liner-to-neck clearance; this topic needs further clarification. Second, kinematic processing of radiographic measurements imparts a risk of radiation exposure. However, we believe that this represents an important data-driven approach to provide feedback on the activities of daily living specific advice for each patient. Third, this study examined only a single component design. Although the design is similar to that of many others that are currently available, the results could differ. Fourth, the sequential motions during squatting were collected into twice, because even the large flat-panel X-ray detector that was used in this study provided a limited field of view (FOV). It might be necessary that the development of fluoroscopy is expected to achieve quite a large FOV. Finally, the patients included in this study were all Japanese with lower BMI compared to the Caucasian average. The patients with a BMI ≥ 30 kg/m^2^ could show different kinematic data with limited maximum hip flexion angle.

## Conclusion

We quantified the change in hip kinematics before and after THA and the liner-to-neck clearance while squatting using the 3D-to-2D model-to-image registration techniques. THA increased the ranges of femoral and hip joint motion and the pelvis tilted anteriorly more after than before THA, with sufficient liner-to-neck clearance during squatting. These data may be beneficial for advising patients after THA regarding postoperative activity restrictions in daily life.

## Abbreviations

2D: Two-dimensional
3D: Three-dimensional
BMI: Body mass index
CT: Computed tomography
DDH: Developmental dysplasia of the hip
FOV: Field of view
HHS: Harris hip score
OA: Osteoarthritis
ROM: Range of motion
SD: Standard deviation
THA: Total hip arthroplasty
